# Anwulignan is a novel JAK1 inhibitor that suppresses non‐small cell lung cancer growth

**DOI:** 10.1111/jcmm.16289

**Published:** 2021-02-01

**Authors:** Xiaomeng Xie, Xiangyu Wang, Xiaodan Shi, Yuanyuan Zhang, Kyle Vaughn Laster, Kangdong Liu, Zigang Dong, Dong Joon Kim

**Affiliations:** ^1^ Department of Pathophysiology School of Basic Medical Sciences Academy of Medical Science College of Medicine Zhengzhou University Zhengzhou HA China; ^2^ China‐US (Henan) Hormel Cancer Institute Zhengzhou HA China; ^3^ The Affiliated Cancer Hospital Zhengzhou University Zhengzhou HA China; ^4^ The Collaborative Innovation Center of Henan Province for Cancer Chemoprevention Zhengzhou HA China; ^5^ International joint research center of cancer chemoprevention Zhengzhou China

**Keywords:** Anwulignan, cell‐derived xenograft, JAK1, NSCLC, STAT3

## Abstract

Anwulignan is a monomer compound derived from Schisandra sphenanthera lignans. It has been reported to possess a spectrum of pharmacological activities, including anti‐bacterial, anti‐inflammatory, anticancer and hepatoprotective properties. However, its anticancer capacity and molecular mechanism(s) against non‐small cell lung cancer (NSCLC) have not been fully elucidated. Anwulignan significantly inhibited cell growth and increased G1‐phase cell cycle arrest in NSCLC cells. Anwulignan strongly attenuates the JAK1/STAT3 signalling pathway by directly targeting JAK1 protein kinase activity *in vitro*. The anticancer activity by Anwulignan is dependent upon the JAK1 protein expression. Remarkably, Anwulignan strongly inhibited tumour growth *in vivo*. In conclusion, Anwulignan is a novel JAK1 inhibitor that may have therapeutic implications for NSCLC management.

## 1 INTRODUCTION

Lung cancer is the most common malignant cancer in the world. 2018 global cancer statistics reported that lung cancer is the first and third leading cause of cancer deaths among males and females, respectively.^1^ Although remarkable advances have been made in the prevention and early treatment of lung cancer, at present the average survival rate of patients with advanced stages of lung cancer is less than 20%.^2^ Non‐small cell lung carcinoma (NSCLC) is the most common type of lung cancer, accounting for more than 80% of all lung cancer patients.^3^ Even though various therapeutics have been studied for NSCLC, poor prognosis and resistance to both radiation and chemotherapy serve as the primary motivation for researchers to identify novel efficacious targets to inhibit NSCLC.^4^, ^5^

The Janus kinase (JAKs) signalling pathways play key roles in the cell proliferation, cell survival and cell invasion.^6^, ^7^ JAKs are a family of non‐receptor tyrosine kinases containing four members: TYK2, JAK1, JAK2 and JAK3.^8^ JAKs are involved in various diseases, including malignancies, through activation of the JAK/STAT signalling pathway.^9^ In addition to its established role as a transcription factor in cancer, signal transducer and activator of transcription 3 (STAT3) regulate mitochondrion functions and gene expression through epigenetic regulation.^10^ When extracellular ligand binds to the cognate cell surface receptor, receptor dimerization and trans‐phosphorylation/activation of JAKs are induced. The activated JAK complex subsequently phosphorylates cytoplasmic receptor‐tails, thereby providing docking sites for STAT (3). STAT3 is phosphorylated on its C‐terminus (Tyr705) and then activated by JAKs.^11^ Among the JAK family kinases, JAK1 has been shown to be the primary driver of STAT3 phosphorylation.^12^ The JAK/STAT pathway promotes tumour proliferation, survival, angiogenesis and tumour metabolism while suppressing antitumour immunity. Therefore, therapeutic inhibition of JAK/STAT signalling pathway offers a considerable benefit.^13^ Increased levels of phosphorylated JAK1 indicated a poor prognosis in NSCLC patient at early stages. The overall survival time for patients with EGFR‐amplified tumours exhibiting increased levels of phosphorylated JAK1 was significantly shortened compared with patients with tumours negative for one or both features.^14^ Additionally, JAK inhibition was shown to impair growth of human K‐RAS‐mutated lung adenocarcinoma.^15^ Although well reported for its functions in tumour proliferation, survival, invasion and immunosuppression,^10^ JAK1/STAT3 signalling also contributes to drug resistance in NSCLC. This observation implies that the JAK1/STAT3 pathway could be a potential therapeutic target for treatment of NSCLC.^16^, ^17^ Inhibition of JAK1/2 signalling was shown to enhance osimertinib’s potency in EGFR‐driven NSCLC xenograft models, including tumours with the therapy‐resistant EGFR T790M mutation.^11^ Taken together, it is considerable that a JAK1 selective inhibitor may enable higher target coverage of the JAK1/STAT3 signalling thereby maximizing the clinical benefit of such agents in therapeutic areas.^18^ Recently, JAK1 inhibitors have been investigated in many clinical trials against cancers,^19^, ^20^ but have achieved only moderate efficacy when applied as monotherapies due to undesired systemic side effects.^6^ Therefore, JAK1 inhibitors combined with additional target inhibitors are under investigation for treatment of various types of cancers.^21^, ^22^

Schisandra sphenanthera, a well‐known Chinese traditional medicine, has historically been used for sedation and hepatoprotection. Anwulignan, a monomer compound derived from Schisandra sphenanthera lignans, is isolated from the ripe fruit of the plant.^20^ Anwulignan has been shown to exhibit various pharmacological properties, including hepatoprotective properties, anti‐bacterial, anti‐inflammatory and anticancer.^23^ Recently, it has also been reported to regulate neuronal survival.^24^ However, anticancer mechanism(s) of Anwulignan have not been investigated in NSCLC. In the present study, we investigated the effects of Anwulignan treatment on the growth of NSCLC cells and tumours and its role in modulating the JAK1/STAT3 signalling pathway.

## 2 MATERIALS AND METHODS

### 2.1 Cell lines

The human NSCLC cell lines A549, H1299, H1650 and H1975 were obtained from the Cell Bank of the Chinese Academy of Sciences (Shanghai, China). Cells were cultured with RPMI‐1640 medium supplemented with 10% foetal bovine serum (FBS; Biological Industries, Cromwell, CT, USA) and 1% antibiotic‐antimycotic and maintained in a 37°C incubator containing 5% CO_2_. Cells were cytogenetically tested and authenticated before generating frozen stocks. Each cell was cultured for a maximum of 8 weeks.

### 2.2 Reagents and antibodies

Anwulignan (purity: > 98% by HPLC) was purchased from ChemFaces (Wuhan, Hunan, China). CNBr‐Sepharose 4B beads were purchased from GE Healthcare (Piscataway, NJ, USA). Antibodies to detect phosphorylated JAK1 (Y1034/Y1035), phosphorylated STAT3 (Y705), phosphorylated AKTs (S473), phosphorylated ERKs (T202/Y204), total JAK1, total STAT3, total AKTs and total ERKs were purchased from Cell Signaling Technology (Beverly, MA, USA). The antibody to detect β‐actin was purchased from Santa Cruz Biotechnology (Santa Cruz, CA, USA). The JAK1 and STAT3 human recombinant proteins for kinase assays or binding assays were purchased from SignalChem (Richmond, BC, Canada).

### 2.3 MTT assay

A549, H1299, H1650 and H1975 cells (2 × 10^3^ cells per well) were seeded for 24 hours in 96‐well plates. Various concentrations of Anwulignan were then added to each well, and cells were incubated for 48 hours. Twenty μl of MTT solution (Solarbio, Beijing, China) was added to each well; the cells were then incubated for 2 hours at 37°C in a 5% CO2 incubator. Next, the cell culture medium was discarded and replaced with 200 µl of DMSO. Formazan crystals were dissolved by gentle agitation. Finally, cell growth was analysed at 570 nm absorbance using the Thermo Multiskan plate‐reader (Thermo Fisher Scientific, Waltham, MA, USA).

### 2.4 Soft agar assay

H1650 and H1975 cells (8 × 10^3^ per well) suspended in growth medium (1640) supplemented with 10% FBS were added to 0.3% agar with or without various concentrations of Anwulignan in a top layer over a base layer of 0.6% agar with or without the same concentration of Anwulignan in each experimental condition. The cultures were maintained at 37°C in a 5% CO2 incubator for 2 weeks. Colonies were imaged under an inverted microscope and quantified with the Image‐Pro Plus software (v.6) program (Media Cybernetics, Rockville, MD, USA).

### 2.5 Foci formation assay

H1650 and H1975 cells (5 × 10^2^ per well) suspended in growth medium (1640) supplemented with 10% FBS were seeded in 6 well plates. Cells were maintained at 37°C in a 5% CO_2_ incubator for 1 week. Foci were subsequently stained with 0.4% crystal violet.

### 2.6 *In vitro* kinase assay

The kinase assay was performed according to the instructions provided by Upstate Biotechnology (Billerica, MA, USA). The recombinant JAK1 (300 ng) protein was mixed with various concentrations of Anwulignan and incubated at room temperature for 15 min. Next, the STAT3 recombinant protein, ATP and 1×buffer were added and incubated at 30°C for 30 minutes. The reaction was stopped by adding 10 μl protein loading buffer. The mixture was subsequently separated by SDS‐PAGE. JAK1 activity was determined using an antibody specific for phosphorylated STAT3 (Y705).

### 2.7 Pull‐down assay using CNBr‐Anwulignan‐conjugated beads

A549 NSCLC cell lysates (1 mg) or recombinant proteins (300 ng) were mixed with Anwulignan‐Sepharose 4B bead or control‐Sepharose 4B beads (50 μl, 50% slurry) at 4°C overnight in reaction buffer (50 mM Tris pH 7.5, 5 mM EDTA, 150 mM NaCl, 1 mM DTT, 0.01% NP40, 2 μg/mL bovine serum albumin). The next day, the mixtures were washed 5 times with buffer (50 mM Tris pH 7.5, 5 mM EDTA, 150 mM NaCl, 1 mM DTT, 0.01% NP40). Binding was determined by Western blotting.

### 2.8 Cell cycle analysis

A549 and H1299 cells (2.5 × 10^4^ or 4 × 10^4^ cells per dish) were seeded in 60‐mm culture dishes for 24 hours. After serum starvation for 24 hours, cells were treated with Anwulignan for 48 hours in growth medium supplemented with 10% FBS. Cells were harvested and fixed in 1 ml of 70% cold ethanol. After rehydration, cells were digested with RNase (100 mg/ml) and stained with propidium iodide (20 mg/ml). The cells were then analysed by flow cytometry.

### 2.9 Establish JAK1 knockdown cells

JAK1 Short hairpin RNA sequences were designed (#2, 5′‐ CCGGGCTCTGGTATGCTCCAAATCGCTCGAGCGATTTGGAGCATACCAGAGCTTTTTG ‐3′; #4, 5′‐ CCGGGGAGAATATCATGGTGGAAGACTCGAGTCTTCCACCATGATATTCTCCTTTTTG ‐3′) and cloned into pLKO.1 lentiviral vector. The pMD2.0G and psPAX2 lentiviral packaging vectors were obtained from Addgene Inc. (Cambridge, MA, USA). Lenti‐X 293T cells were transfected with each viral vector and packaging vectors using Lipofectamine 2000 (Invitrogen, Grand Island, NY, USA) and incubated for 48 h. Virus particle‐containing media were collected and filtered using a 0.45 µm sodium acetate syringe filter. The collected cell culture media were mixed with 8 μg/ml of polybrene (Millipore, Billerica, MA, USA) and then applied to A549 or H1975 cells for 48 hours. After selection with puromycin (0.75‐1 μg/ml) for 48 h, cells were used for further study.

### 2.10 Cell‐derived lung cancer xenografts

To examine the effect of Anwulignan on cell‐derived lung cancer xenograft (CDX) tumour growth, 8 female nude mice (6‐week‐old, 4 mice per Anwulignan treatment group) were purchased from the Chinese Academy of Sciences (Beijing, China). H1975 cells (1 × 10^7^ cells/0.1 ml per mouse) were resuspended in PBS and injected subcutaneously into the lower back of the mice. Tumours were allowed to form over a period of 2 weeks. Tumour volume was measured with calipers and calculated according to the formula, V = 0.52 × (length × width × height) twice per week. Mice were monitored until tumours reached 1.5 cm^3^ total volume. Finally, mice were euthanized and body weight of each mouse was determined before the tumours and organs were harvested for further analysis.

### 2.11 Haematoxylin‐eosin (HE) staining and immunohistochemistry (IHC)

Tumour tissues were prepared for IHC. Liver, spleen and kidney were prepared for H&E analysis. Tissues were embedded in paraffin and sectioned onto slides. The slides were baked at 65°C for 3 hours. After de‐paraffinization and hydration, slides were boiled in citrate buffer for 90 seconds at a high temperature and pressure. Slides were then treated with H_2_O_2_ for 5 minutes, and incubated with primary antibody at 4°C overnight. After incubation with a secondary antibody, slides were stained with DAB (3, 3'‐diaminobenzidine). The IHC staining was quantitated by calculating the integrated optical density (IOD) value measured by Image‐Pro Plus.

### 2.12 *In vivo* toxicity assay

Twelve female nude mice (6‐weeks old, 4 mice per group) were maintained under ‘specific pathogen‐free’ conditions. Mice were divided into 3 groups as follows: 1) vehicle group; 2) 20 mg Anwulignan/kg of body weight in vehicle ; 3) 40 mg Anwulignan /kg of body weight in vehicle. Mice were orally treated with Anwulignan or vehicle (10% DMSO in 20% tween 80) for 2 weeks. Blood samples were collected, and AST or ALT activity from serum was detected at 510 nm.

### 2.13 Statistical analysis

All quantitative results are expressed as mean values ± SD or ± SE Significant differences (*P* value < 0.05) were compared using the Student’s t test or one‐way analysis of variance (ANOVA).

## 3 RESULTS

### 3.1 Anwulignan inhibits NSCLC cell growth

**FIGURE 1:**
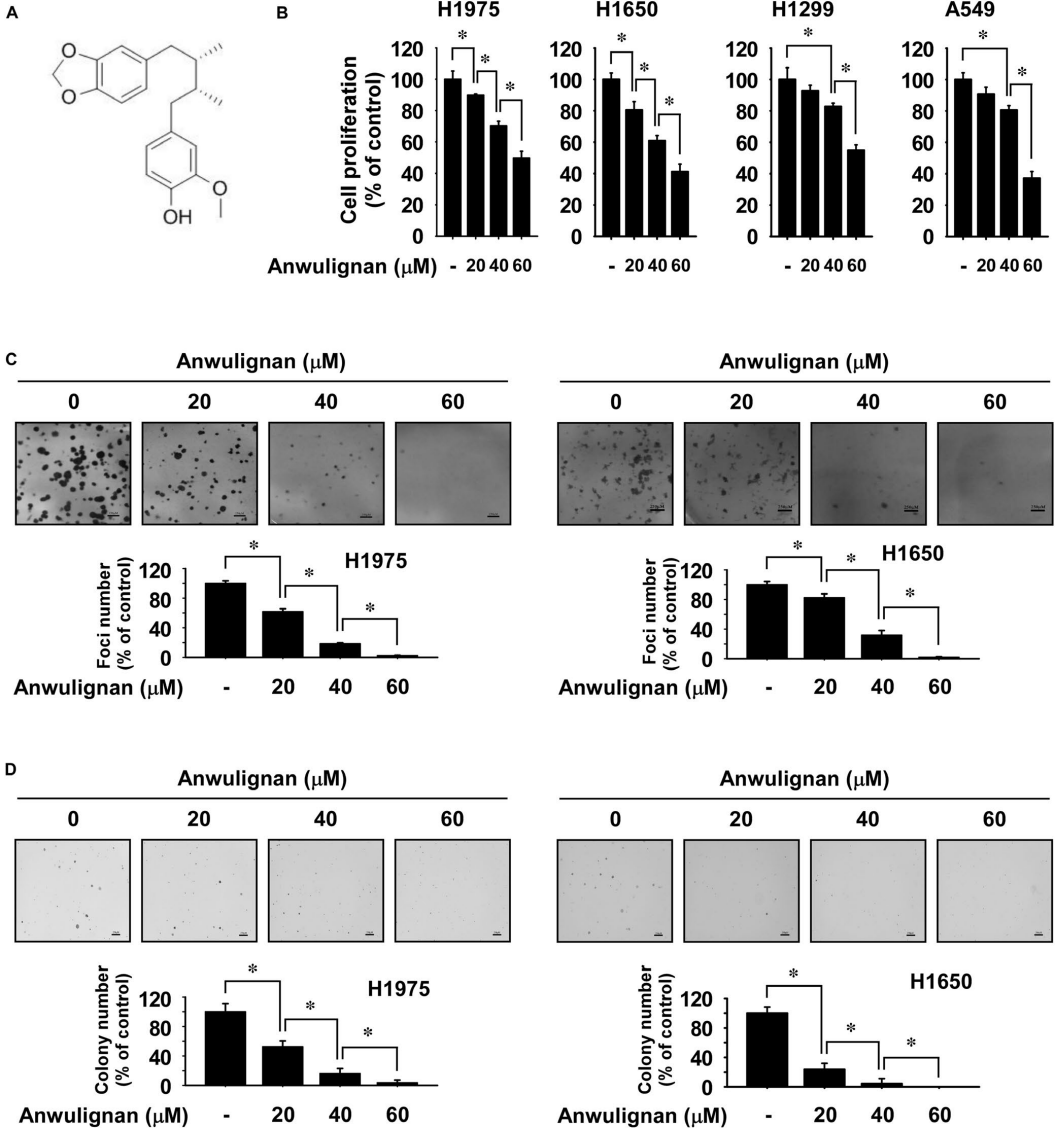
Anwulignan inhibits NSCLC cell growth. A, Chemical structure of Anwulignan. B, Effect on the growth of NSCLC cells by Anwulignan treatment. Cell growth was analysed by the MTT assay. C, Effect on foci formation by Anwulignan treatment. Foci ability was determined by the foci formation assay. D, Effect on anchorage‐independent NSCLC cell growth by Anwulignan treatment. Colonies were imaged using a microscope and quantified with the Image‐Pro PLUS (v.6) computer software program. For B–D, data are shown as means ± SD of values from 3 independent experiments each with triplicate samples. One‐way ANOVA with Tukey's honestly significant difference (HSD) post hoc test was used to analyse the data. The asterisk (*) indicates a significant (*P* < 0.05) inhibitory effect of Anwulignan

Anwulignan is a 4‐(2S,3R)‐4‐(1,3‐benzodioxol‐5‐yl‐2‐3‐dimetybty]‐2‐methoxyphenol compound (Figure 1A). To examine the effect of Anwulignan on NSCLC cell growth and foci formation ability, cells were treated with Anwulignan at several concentrations for 48 h or 10 days, and cell growth was analysed by MTT assay or foci formation assay, respectively. The results indicated that NSCLC cell growth and foci number were suppressed by Anwulignan treatment in a dose‐dependent manner (Figure 1B and C). Additionally, Anwulignan significantly inhibited anchorage‐independent growth of NSCLC cells (Figure 1D).

### 3.2 Anwulignan induces G1‐phase cell cycle arrest in NSCLC cells

**FIGURE 2:**
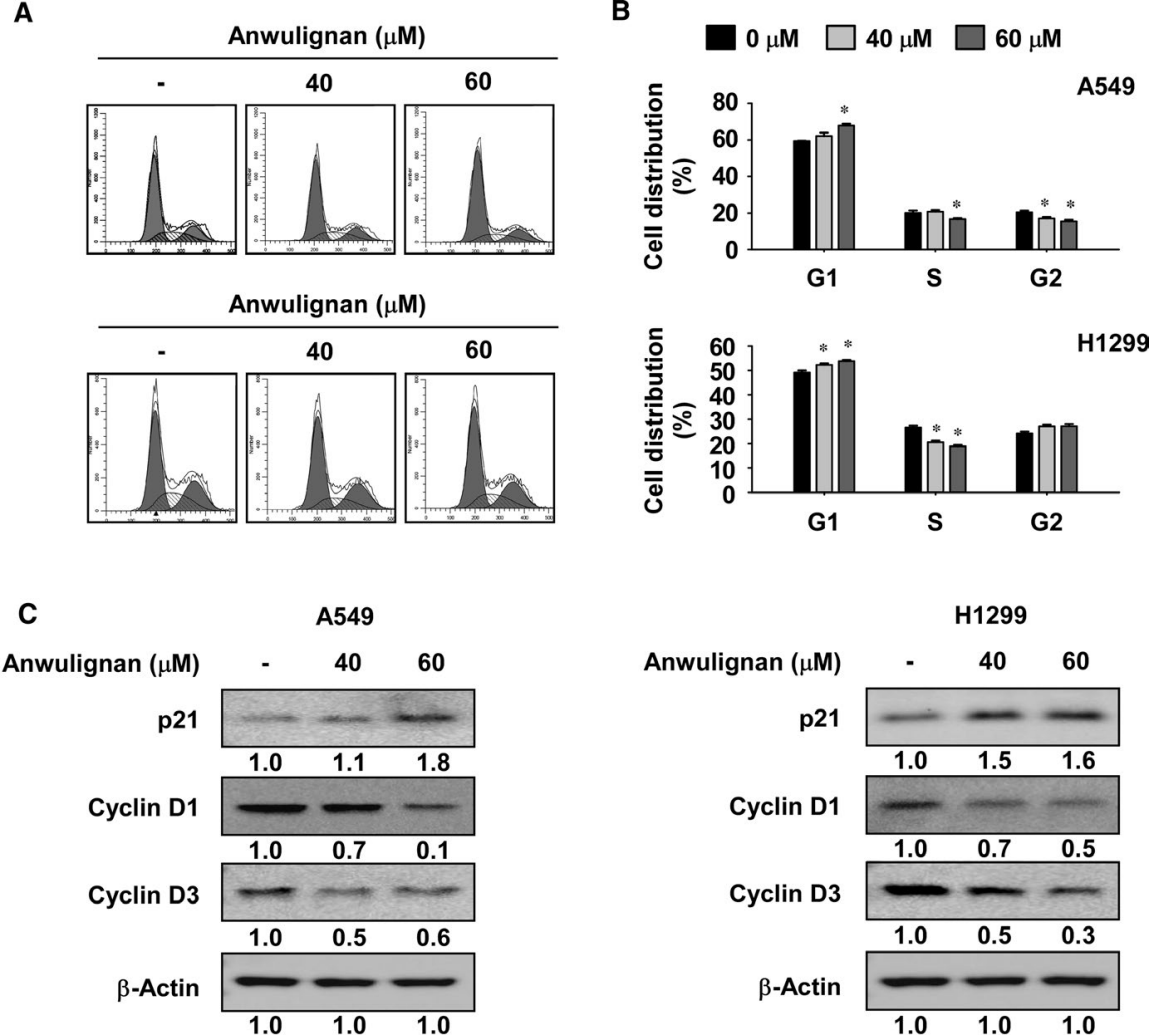
Anwulignan induces G1‐phase cell cycle arrest. A, B, Effect of Anwulignan on cell cycle in A549 and H1299 NSCLC cells was examined by Fluorescence‐Activated Cell Sorting (FACS). Data are indicated as means ± SD of values from 3 independent experiments. One‐way ANOVA with Dunnett’s post hoc test was used to analyse the data. The asterisk (*) indicates a significant (*P* < 0.05) difference between Anwulignan‐treated cells and DMSO‐treated cells. C, Effect of Anwulignan on the expression of cell cycle marker proteins was examined by Western blotting. Band density was measured using the Image J (NIH) software program

To determine whether Anwulignan could affect cell cycle regulation, we performed cell cycle analysis by flow cytometry. After serum starvation for 24 hours, A549 and H1299 NSCLC cells were treated with various concentrations of Anwulignan for 48 hours in growth medium (10% FBS). Results indicated that Anwulignan treatment increased G1‐phase cell cycle arrest in NSCLC cells (Figure 2A and B). Furthermore, to explore the underlying mechanism, we detected the expression of cell cycle marker proteins by Western blotting. Results showed that Anwulignan consistently inhibited the expression of cyclin D1 and cyclin D3 and increased the expression of p21 in both cell lines (Figure 2C).

### 3.3 Anwulignan is a novel JAK1 inhibitor

**FIGURE 3:**
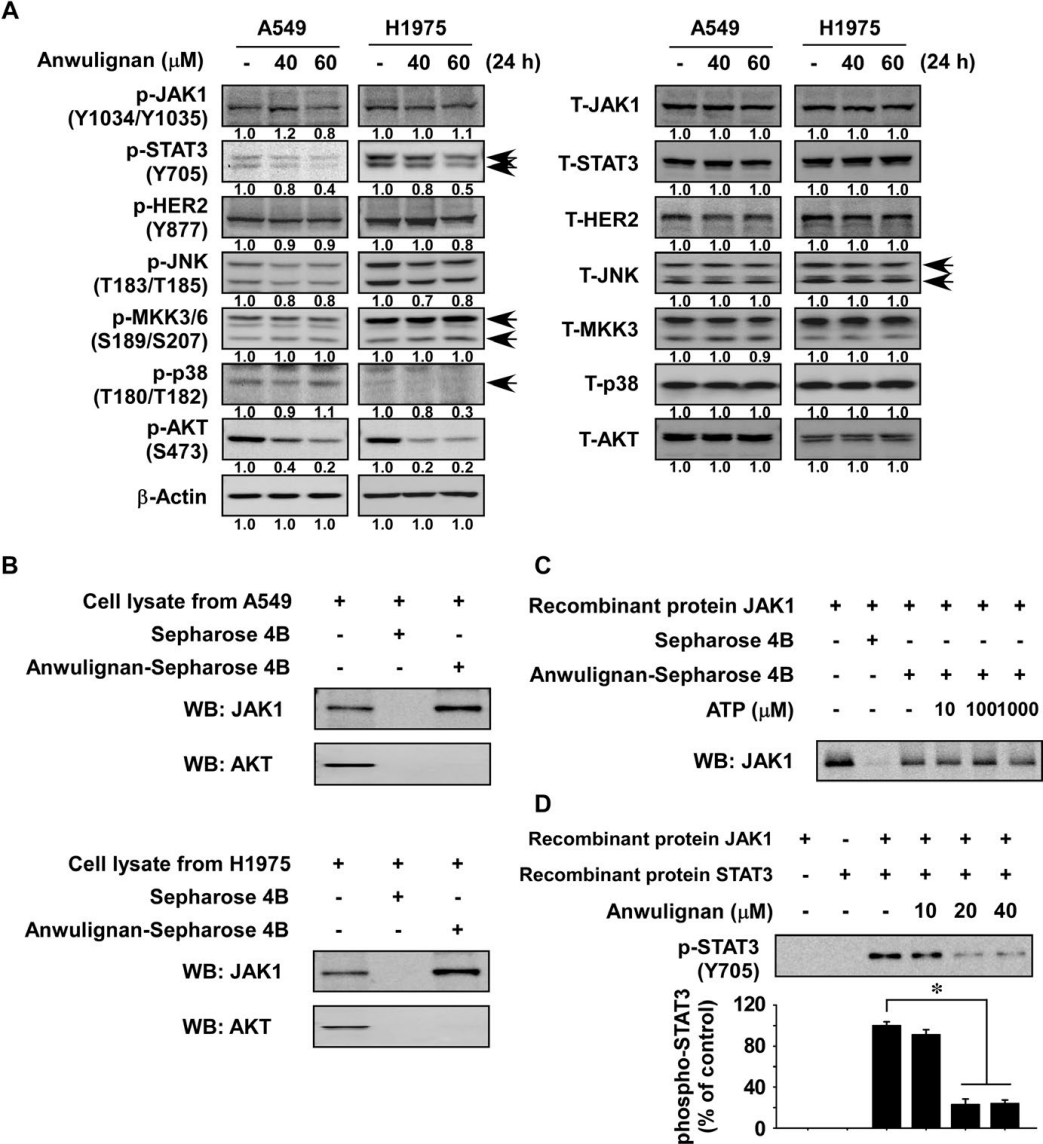
Anwulignan strongly inhibits the JAK1/STAT3 signalling pathway. A, Effect of Anwulignan on various kinase signalling in NSCLC cells. Cells were treated with Anwulignan for 24 hours and then various signalling molecules were examined by Western blotting. Anwulignan directly binds to JAK1 in (B) an NSCLC cell lysate or (C) recombinant proteins. The cell lysate or recombinant protein was incubated with Anwulignan‐conjugated Sepharose 4B beads or with Sepharose 4B beads alone. Pulled down proteins were analysed by Western blotting. D, Effect of Anwulignan on JAK1 kinase activity was measured by an *in vitro* kinase assay. For all experiments, similar results were detected from three independent experiments. The asterisk (*) indicates a significant (*P* < 0.05) inhibitory effect of Anwulignan

To identify potential molecular targets of Anwulignan, we examined the effect of Anwulignan treatment on various signalling molecules in A549 and H1975 NSCLC cells. Results indicated that Anwulignan strongly inhibited phosphorylated STAT3 expression, whereas other signalling proteins were marginally affected (Figure 3A). Next, we performed an *in vitro* binding assay to confirm direct binding of Anwulignan and JAK1 using NSCLC cell lysate. Results indicated that Anwulignan directly binds with JAK1 but not AKT (Figure 3B). To explore whether Anwulignan could interact with JAK1 at its ATP pocket, we performed a binding assay using recombinant JAK1 protein with different concentration of ATP. The results suggested that Anwulignan was not an ATP competitor (Figure 3C). To examine whether Anwulignan could regulate JAK1 activity, we performed *in vitro* kinase assays using a recombinant JAK1 protein and a recombinant STAT3 protein. The findings showed that Anwulignan inhibited the phosphorylation of STAT3 in a dose‐dependent manner by directly targeting JAK1 (Figure 3D). Additionally, to determine whether the activities other signalling proteins are affected by Anwulignan, the activities of various protein kinases were quantified (Eurofins, https://www.eurofins.com). ABL, AKT1, AMPKα1, AURKA, BRAF, CDK2/CCNE, CDK4/CCND3, CHEK1, EGFR, DN‐PK, ERBB2, ERK1, FAK, FGFR, VEGFR1, FYN, GSK3β, HIPK1, IKKα, KIT, MEK1, MET, MKK6, MSK1, mTOR, NEK1, p70S6K, PAK4, PDK1, PI3K, PIM1, PKCα, RSK2, SRC, TBK1 or TLK1 kinase and their respective substrates were incubated with or without Anwulignan. The results showed that the activities of these kinases were marginally affected by Anwulignan (Figure S1A and B).

### 3.4 JAK1 knockdown suppresses NSCLC cell growth

To examine the expression levels of JAK1 and STAT3 proteins in NSCLC cells, we performed Western blotting. The results indicated that JAK1 phosphorylation was up‐regulated in A549 and H1975 cells compared to H1299 and H1650 cells (Figure S2); thus, A549 and H1975 cells were used for further study. To examine the effect of JAK1 knockdown (KD) on NSCLC cell growth, we established stable shRNA control and JAK1 KD cell lines and detected the expression level of JAK1 protein. The finding indicated that phosphorylated and total JAK1 proteins were strongly decreased in shJAK1 #2 and shJAK1 #4 cells (Figure S3).

**FIGURE 4:**
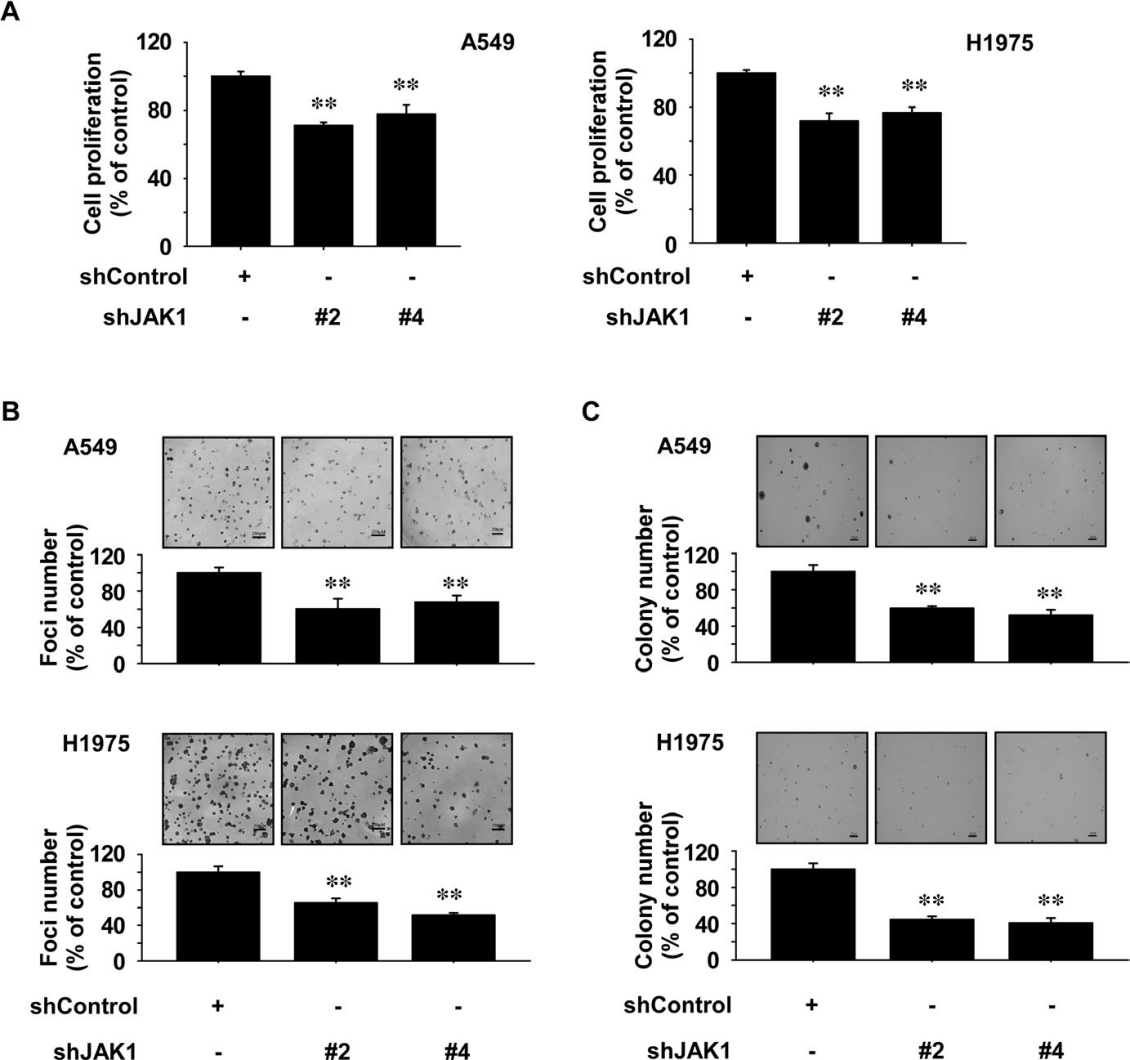
JAK1 is a therapeutic target in NSCLC cells. A, Effect of JAK1 knockdown on NSCLC cell growth was determined by MTT assay. B, Effect of JAK1 knockdown on foci formation ability of NSCLC cells. Cells were incubated for 1 week and then foci number was counted. C, Effect on anchorage‐independent growth of NSCLC cells by JAK1 knockdown. After incubation for 2 weeks, colony number was counted. For all, data are shown as means ± SD of values from 3 independent experiments. One‐way ANOVA with Dunnett’s post hoc test was used to analyse the data. The asterisk (*) indicates a significant (*P* < 0.05) inhibitory effect of JAK1 knockdown

To examine effect on anchorage‐dependent or ‐independent growth of NSCLC cells by JAK1 knockdown, we performed MTT, soft agar assay or foci formation assays. Findings indicated that anchorage‐dependent and ‐independent growth of NSCLC cells was strongly inhibited by JAK1 knockdown (Figure 4A‐C).

### 3.5 The inhibitory effect of Anwulignan is dependent on the JAK1 expression

**FIGURE 5:**
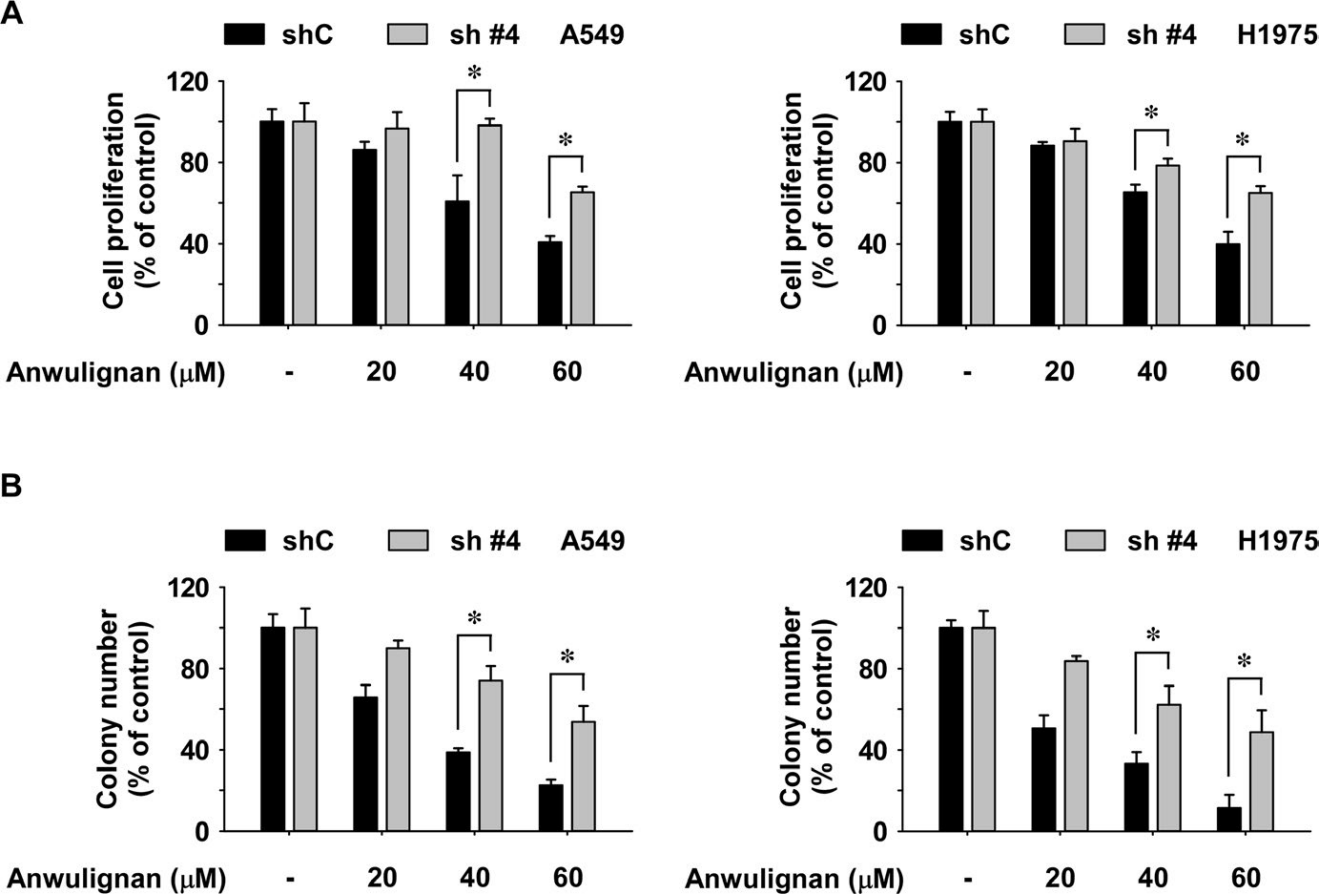
JAK1‐dependent anticancer activity of Anwulignan. A, B, JAK1‐dependent inhibitory effect of Anwulignan on anchorage‐dependent (A) and anchorage‐independent (B) NSCLC cell growth. shJAK1 or shControl cells were treated with or without with Anwulignan for 48 hours or 2 weeks, respectively. Cell growth was analysed by the MTT assay and soft agar assay. For all experiments, data are shown as means ± SD of values from 3 independent experiments. An un‐paired *t* test was used to analyse the data. The asterisk (*) indicates a significant effect (*P* < 0.05) of Anwulignan treatment between JAK1 knockdown cells and shControl cells

To determine the JAK1‐dependent anticancer effect of Anwulignan, shControl or shJAK1 cells were treated with Anwulignan. NSCLC cell growth was examined by MTT or soft agar assay. The results showed that shJAK1 cells were resistant to Anwulignan’s effect on anchorage‐dependent or ‐independent cell growth compared to cells expressing shControl (Figure 5A and B).

### 3.6 Anwulignan reduces H1975 cell‐derived xenograft (CDX) tumour growth *in vivo*

**FIGURE 6:**
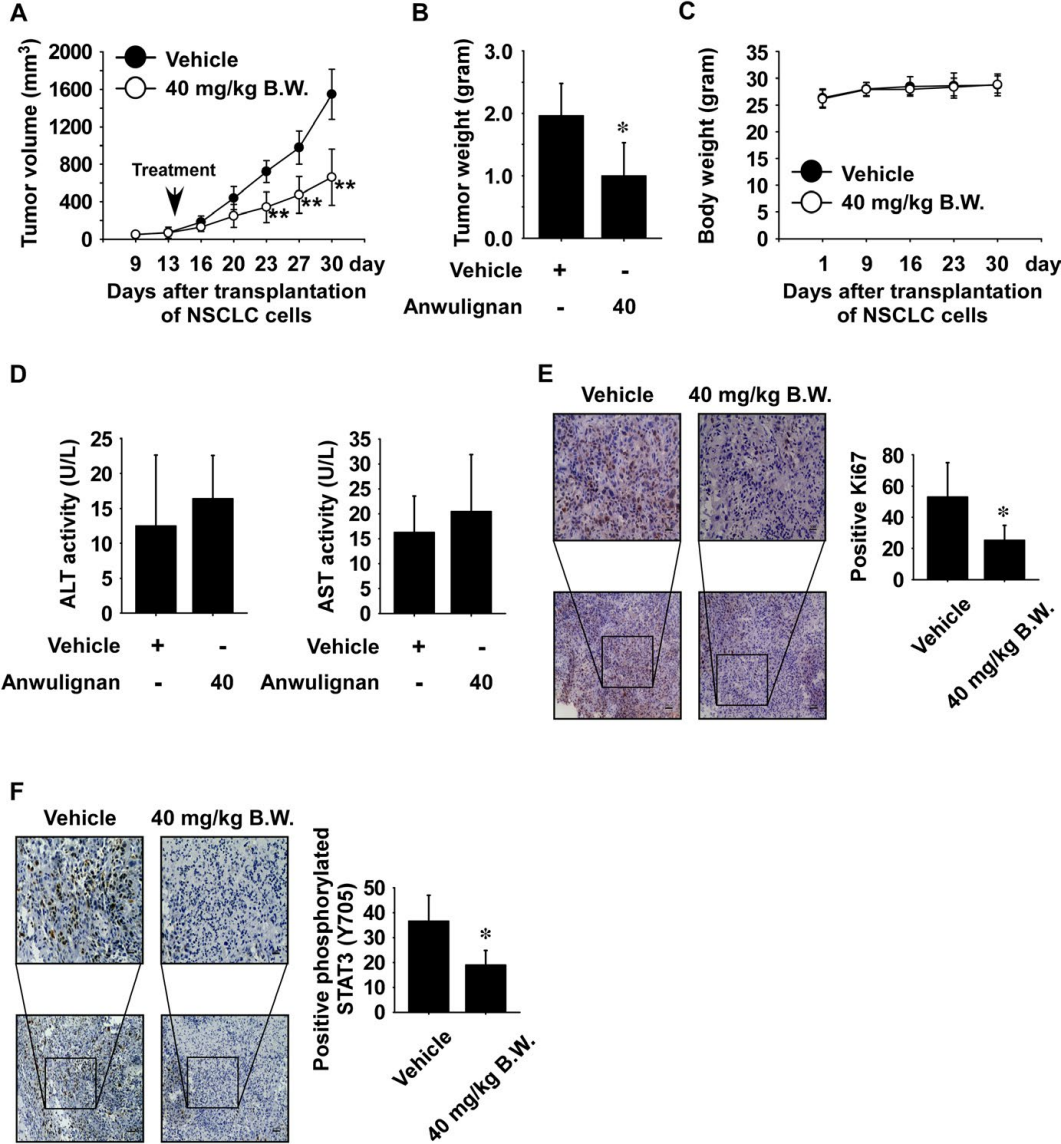
Anwulignan reduces H1975 cell‐derived xenograft tumour growth *in vivo*. The effect of Anwulignan on H1975 cell‐derived xenograft tumour growth was assessed. Tumour‐bearing mice (vehicle group or 40 mg/kg of Anwulignan group) were orally treated with Anwulignan or vehicle for 18 days. Effect of Anwulignan on (A) tumour volumes, (B) tumour weight and (C) mice body weight was measured on the indicated days. D, The effect of Anwulignan on the activity of AST or ALT was analysed. All data are shown as mean ± SE from each group. E, Anwulignan inhibits expression of Ki‐67 protein. F, Anwulignan reduces phosphorylated STAT3 expression. Non‐parametric test was used to analyse the data. The asterisk (*) indicates a significant (*P* < 0.05) inhibitory effect of Anwulignan treatment

To determine the toxicity of Anwulignan *in vivo*, mice were orally treated with Anwulignan at 20, 40 mg/kg or vehicle for 2 weeks. The results showed that the alanine transaminase (ALT) and aspartate transaminase (AST) activity were not significantly altered in the Anwulignan‐treated groups (20 and 40 mg/kg) compared with the vehicle‐treated group (Figure S4A and B). Therefore, we used 40 mg/kg Anwulignan for the *in vivo* mouse study. To determine whether Anwulignan could inhibit NSCLC tumour growth *in vivo*, we established H1975 cell‐derived xenografts in mice. Mice were orally administrated vehicle or 40 mg/kg Anwulignan 5 times per week over a period of 27 days. The results indicated that Anwulignan significantly inhibited the tumour volume and weight relative to the vehicle‐treated group (Figure 6A and B). To determine the potential toxicity of Anwulignan on the mice, the body weight of each mouse was measured. Additionally, ALT and AST activity were measured. Results showed that body weight, ALT activity and AST activity were not significantly changed in mice treated with Anwulignan at 40 mg/kg compared with the vehicle‐treated group (Figure 6C and D). Additionally, the liver, spleen and kidney tissues were extracted and stained with haematoxylin and eosin (H&E). Results showed no distinct morphological changes in the both group mice (Figure S5A‐C). To examine effect of Anwulignan on Ki‐67, a proliferation marker protein, we performed immunohistochemistry. Results showed that the Ki‐67 expression was strongly reduced in Anwulignan‐treated group (Figure 6E). To determine whether Anwulignan could inhibit the expression of STAT3, CDX tumour tissues were analysed by immunohistochemistry. Results showed that phosphorylated STAT3 was strongly decreased in the Anwulignan‐treated group (Figure 6F).

## 4 DISCUSSION

NSCLC is a remarkably urgent public health issue. Although several FDA‐approved target therapies are currently used in clinical settings, none of these therapies are uniformly effective in all patient groups.^24^ Thus, identification of novel therapies is of utmost importance to research scientists. Anwulignan is a monomer compound derived from Schisandra sphenanthera lignans.^20^ Recently, Schisandra sphenanthera extract has been reported to be effective in protecting the liver through activation of the Nrf2‐mediated defence response.^25^, ^26^ However, the molecular targets of Anwulignan and its potential therapeutic effects have not been elucidated in NSCLC. Here, we reported that Anwulignan suppresses non‐small cell lung cancer growth by directly targeting JAK1 *in vitro* and *in vivo*.

Based on the results of signalling pathway and *in vitro* assays, we identified the molecular target of Anwulignan as JAK1 (Figure 3). JAK1 is a member of Janus kinases that can specifically phosphorylate and activate STAT3.^11^ Previously, the JAK1/STAT3 signalling pathway was implicated as an oncogene in NSCLC.^27^, ^28^ STAT3 is activated in NSCLC patients and induces NSCLC cell growth.^29^, ^30^ Therefore, our findings suggest that Anwulignan can inhibit NSCLC cancer growth by inhibiting the JAK1/STAT3 signalling pathway. Additionally, inhibition of STAT3 by JAK1 inhibitor or JAK2 inhibitor could overcome EGFR‐TKI resistance in human NSCLC.^31^ The IL‐6/JAK1 pathway was reported to drive PD‐L1 Y112 phosphorylation to promote cancer immune evasion.^32^ FGF receptor and JAK kinases led to autocrine activation of STAT3, consequently promoting drug resistance.^33^ Therefore, Anwulignan could be a potential therapy used to treat drug‐resistant NSCLC alone or in combination with other known therapeutic drugs.

To verify the oncogenic role of JAK1 in NSCLC, A549 and H1975 cells depleted of JAK1 were established through knockdown. The results of the cell proliferation assays suggested that JAK1 could be a potential therapeutic target (Figure 4). We also examined whether the inhibitory effect of Anwulignan is dependent on the JAK1 expression. We found that JAK1 knockdown cells were resistant to Anwulignan's inhibitory effect on proliferation compared to shControl cells (Figure 5A and B). Overall, we suggest that the effect of Anwulignan on the NSCLC cell growth is dependent on JAK1 expression. However, the result of the inhibitory effect of Anwulignan in cells with low JAK1 expression (shJAK1 #4) suggested that 60 μM of Anwulignan still reduced cell growth (Figure 5A and B). Therefore, we also suggest that there are additional molecular targets of Anwulignan. To explore the other potential targets of Anwulignan, we performed cancer‐related kinase screening; however, we were unable to identify additional targets within our kinase panel (Figure S1A and B). Therefore, we suggest that JAK1 is the main target protein of Anwulignan in NSCLC cells. In addition, JAKs family contain a JAK homology (JH)1 domain. Full activation of JAKs results from disrupting JH2‐mediated inhibition of JH1 kinase activity; activated JAK enzymes subsequently serve as docking sites for STAT3 proteins.^34^ As the JAKs family share considerable structural similarity, further studies associated with other members of the JAKs family—JAK2, JAK3 and TYK2, are planned to investigate the additional targets of Anwulignan.

Previous research reported that JAK1/STAT3 signalling pathway was regulated by several growth factors, and subsequently contributes to cancer progression.^35^ Inhibitors that are able to attenuate JAK1/STAT3 signalling have been shown to suppress cell proliferation of lymphoma and oesophageal cancer.^36^, ^37^ This observation may in part be due to the importance of JAK1/STAT3 signalling in facilitating G1 to S phase cell cycle transition through regulation of cyclin D1 and p21 expression.^38^, ^39^ Therefore, we suggest that the inhibition of NSCLC cell growth by Anwulignan is correlated with G1 phase cell cycle arrest due to a reduction in cyclin D1 and cyclin D3 expression and an induction in p21 expression.

To explore the *in vivo* effect and toxicity of Anwulignan, we established cell‐derived xenograft mice models using lung cancer cell lines. We first examined the toxicity of Anwulignan in an *in vivo* mouse model (Figure S4A and B) and CDX mice (Figure 6D) by measuring ALT and AST activities in serum after Anwulignan treatment. The results indicated that there was no significant difference between the Anwulignan‐treated group and the vehicle‐treated group. We next investigated the antitumour effect of Anwulignan on NSCLC tumour growth and the results indicated that Anwulignan significantly suppressed tumour growth by reducing the level of phosphorylated STAT3 (Figure 6F). Additionally, JAK1 activity is positively correlated with Ki‐67 expression.^40^, ^41^ Therefore, we investigated whether Anwulignan could inhibit Ki‐67 expression in NSCLC tumour tissues. Results indicated that Anwulignan strongly reduced the Ki‐67 expression (Figure 6E). Therefore, targeting JAK1 protein with Anwulignan may provide therapeutic effects against NSCLC.

In conclusion, our study provides evidence that Anwulignan can inhibit NSCLC progression *in vitro* and *in vivo* through direct targeting of JAK1.

## CONFLICT OF INTEREST

None of the authors have any competing interests.

## AUTHOR CONTRIBUTIONS

X.X.: preparing the manuscript and performed the *in vitro*, the cell‐based and *in vivo* experiments; X.W., X.X and Y. Z.: assisting the cell‐based assays; K.L. and K. L.: performing the data analysis and manuscript editing; Z.D. and D.J.K.: supervising the overall experimental design and providing the idea.

## Supporting information

Supplementary Material

## Data Availability

All data in this study are available if requested
