# Supplementary material for: Anwulignan is a novel JAK1 inhibitor that suppresses non‐small cell lung cancer growth
Source: J Cell Mol Med. 2021 Feb 1;25(5):2645–54. doi: 10.1111/jcmm.16289 (PMC7933975; doi:10.1111/jcmm.16289)
Supplement: Supplementary file 1 — Supplementary Material [file JCMM-25-2645-s001.zip › jcmm16289-sup-0006-FigLegends.docx]

**Supplemental Figure 1. Effect of Anwulignan on 37 cancer-related kinases.** (A, B) Effect of Anwulignan on the kinase activity of ABL, AKT1, AMPKα1, AURKA, B-RAF, CDK2/CCNE, CDK4/CCND3, CHEK1, DNA-PK, EGFR(L858R), EGFR(T790M), ERBB2, ERK1, FAK, FGFR1, VEGFR1, FYN, GSK3β, HIPK1, IKKα, KIT, MEK1, MET, MKK6, MSK1, mTOR/FKBP12, NEK1, p70S6K, PAK4, PDK1, PI3K(p110α/p65α), PIM1, PKCα, RSK2, TBK1 and TLK1. An *in vitro* kinase assay was conducted with each kinase and their respective substrates in the presence or absence of Anwulignan. Data are shown as means ± S.D. of duplicate values. (C) Effect of Anwulignan on the phosphorylation of MET, EGFR, and cABL in A549 and H1975 NSCLC cells. Cells were treated with Anwulignan for 24 h and the kinases were analyzed by Western blotting. Similar results were observed from 3 independent experiments. Band density was measured using the Image J (NIH) software program.

**Supplemental Figure 2. Expression of JAK1 and STAT3 in non-small cell lung cancer cells.** Expression JAK1 signaling proteins in NSCLC cells was analyzed by Western blotting. Similar results were observed from 3 independent experiments. Band density was measured using the Image J (NIH) software program.

**Supplemental Figure 3. Effect of JAK1 knockdown or shControl on JAK1/STAT3 signaling in A549 or H1975 NSCLC cells.** Expression of JAK1 and STAT3 in JAK1 knockdown or shControl cells was determined by Western blotting. Similar results were observed from 3 independent experiments. Band density was measured using the Image J (NIH) software program.

**Supplemental Figure 4. Anwulignan has minimal liver toxicity *in vivo*.** Effect of Anwulignan on the activity of AST or ALT was assessed. AST and ALT activity were calculated using Anwulignan-treated or vehicle-treated mice. All data are shown as mean ± S.E. of values obtained from each group (n = 4)

**Supplemental Figure 5. Anwulignan has minimal liver, kidney, and spleen toxicity *in vivo*.** Representative images of showing hematoxylin-eosin stained liver (A), kidney (B) and spleen (C) tissues excised from Anwulignan-treated mice.
