# Supplementary figures and images for: Anwulignan is a novel JAK1 inhibitor that suppresses non‐small cell lung cancer growth
Source: J Cell Mol Med. 2021 Feb 1;25(5):2645–54. doi: 10.1111/jcmm.16289 (PMC7933975; doi:10.1111/jcmm.16289)

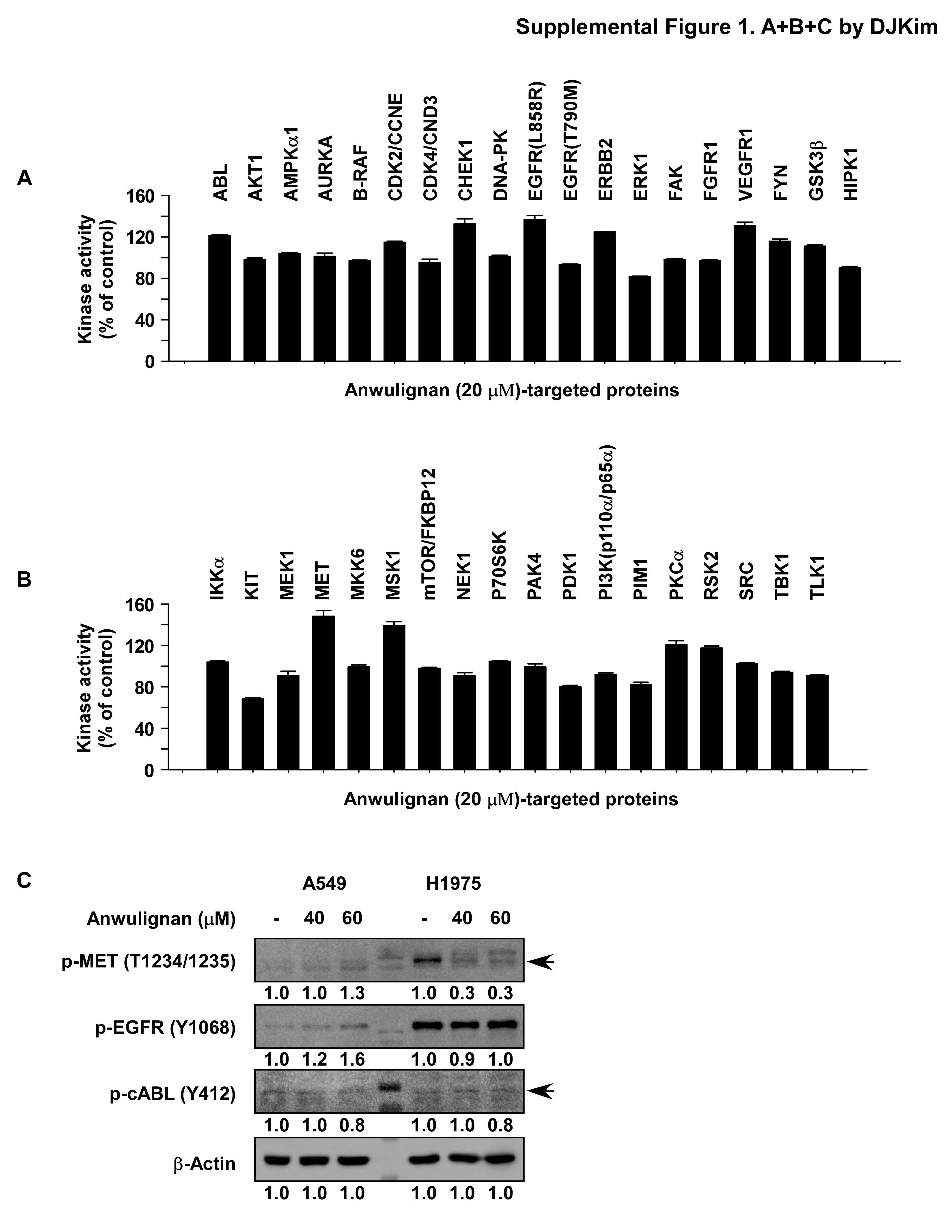

Supplement: Supplementary file 1 — Supplementary Material [file JCMM-25-2645-s001.zip › jcmm16289-sup-0001-FigS1.jpg]

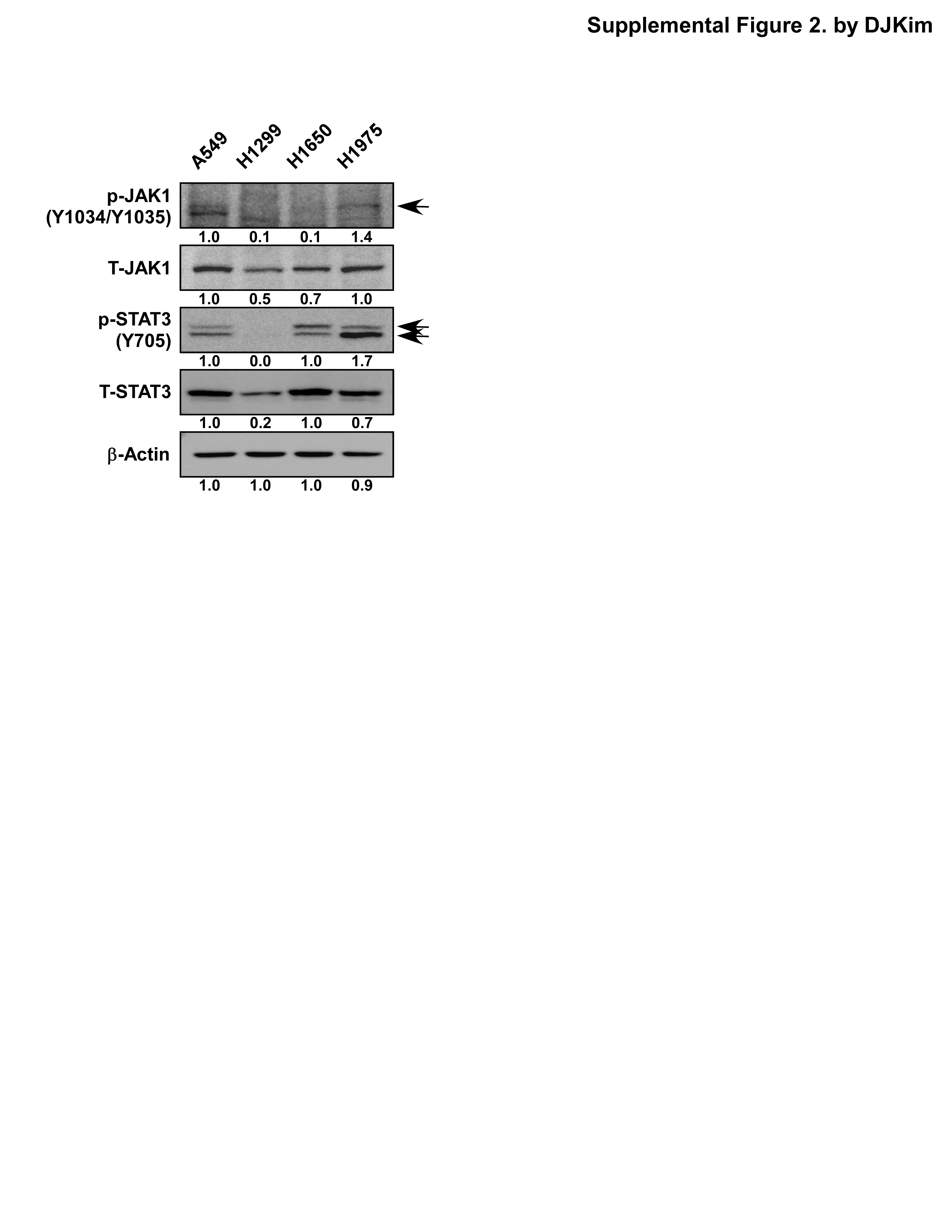

Supplement: Supplementary file 1 — Supplementary Material [file JCMM-25-2645-s001.zip › jcmm16289-sup-0002-FigS2.jpg]

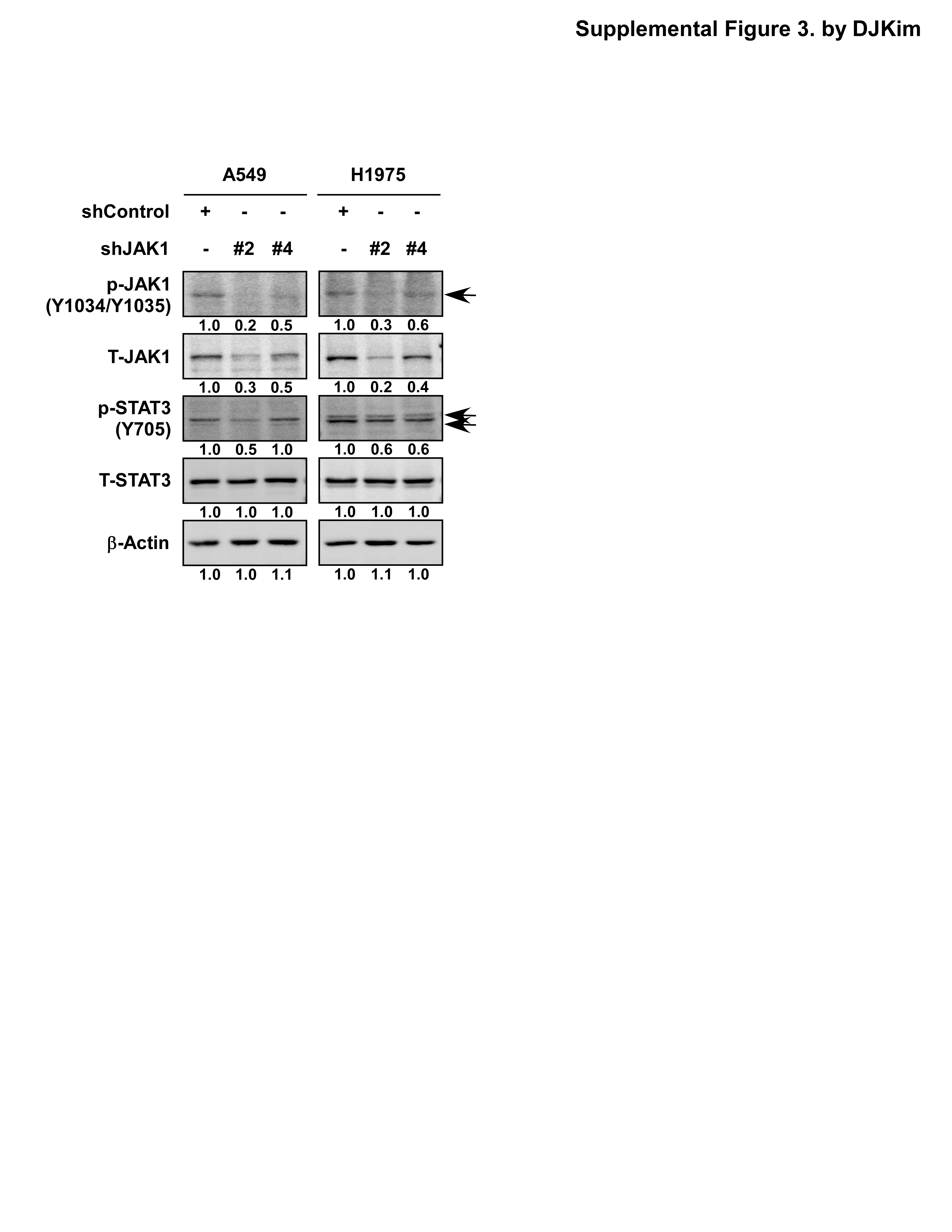

Supplement: Supplementary file 1 — Supplementary Material [file JCMM-25-2645-s001.zip › jcmm16289-sup-0003-FigS3.jpg]

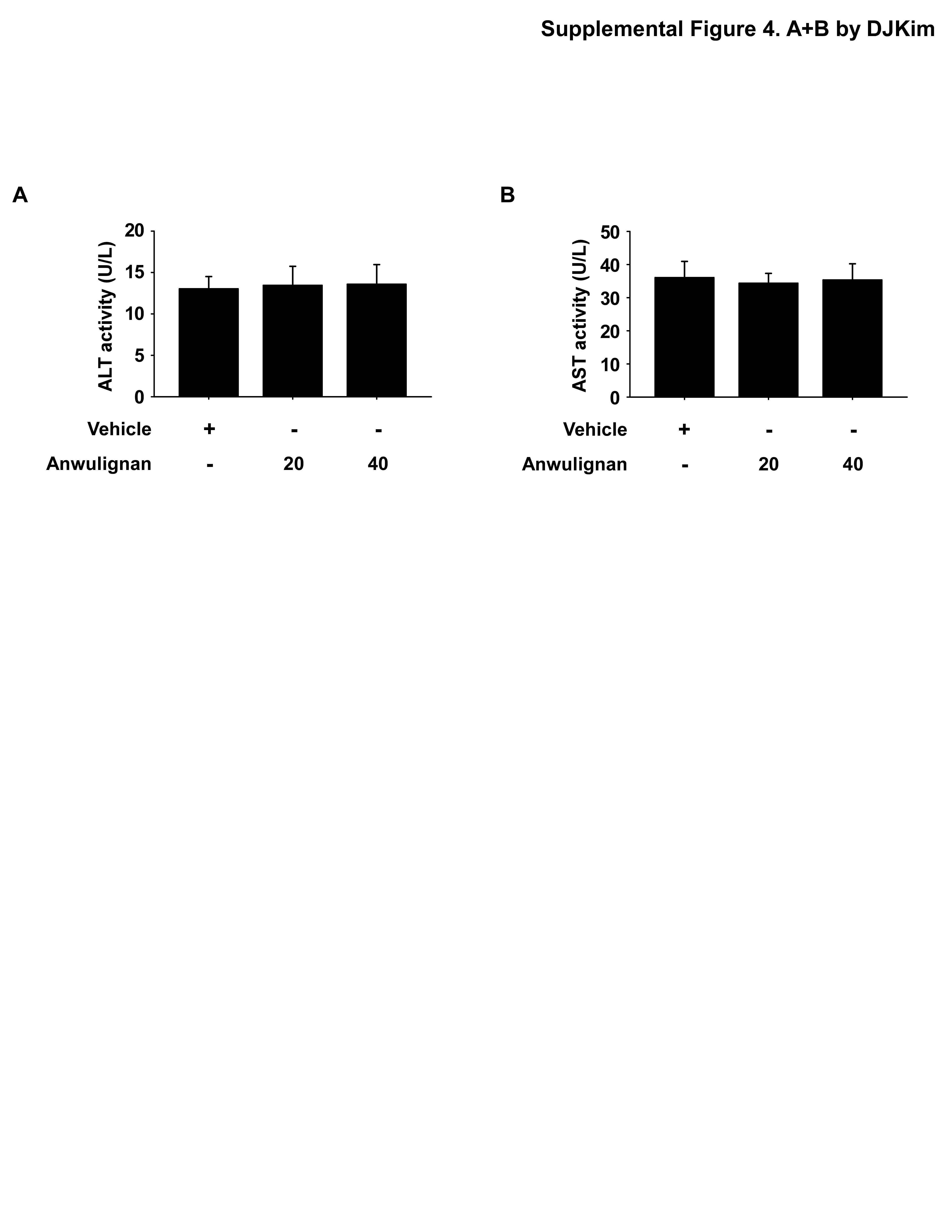

Supplement: Supplementary file 1 — Supplementary Material [file JCMM-25-2645-s001.zip › jcmm16289-sup-0004-FigS4.jpg]

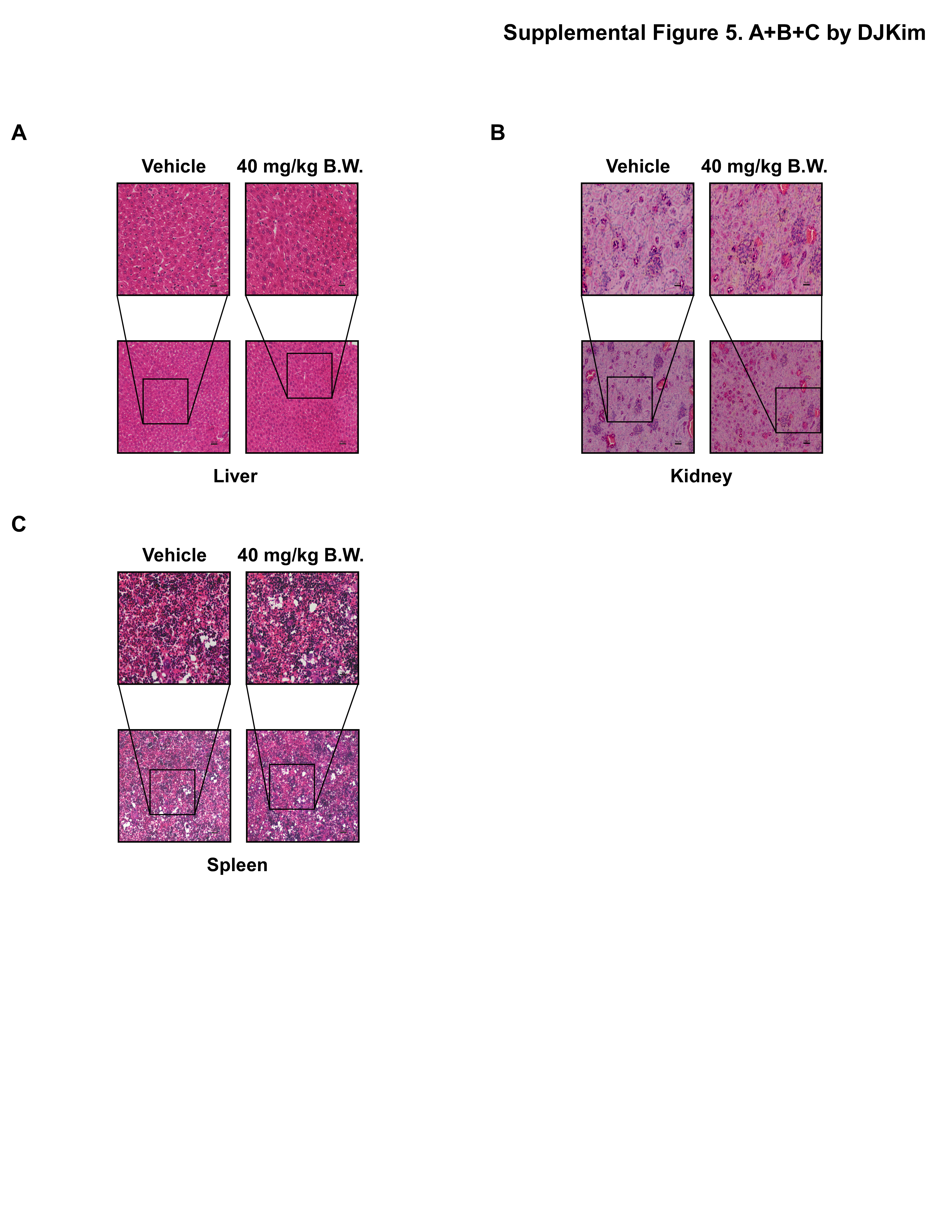

Supplement: Supplementary file 1 — Supplementary Material [file JCMM-25-2645-s001.zip › jcmm16289-sup-0005-FigS5.jpg]
